# Perches used as environmental enrichment influence fast-growth broilers’ biomechanics and locomotor morphometry at the age of 42 days

**DOI:** 10.1371/journal.pone.0313214

**Published:** 2024-11-14

**Authors:** Aerica Cirqueira Nazareno, Robson Mateus Freitas Silveira, Danielle Priscila Bueno Fernandes, Jessica Chierri, Luiz Otavio Pradella, Iran José Oliveira da Silva

**Affiliations:** 1 Environment Livestock Research Group (NUPEA), “Luiz de Queiroz” Agriculture School (ESALQ), Department of Biosystems Engineering, University of São Paulo (USP), Piracicaba, São Paulo State, Brazil; 2 Department of Animal Science, “Luiz de Queiroz” Agriculture College (ESALQ), University of São Paulo (USP), Piracicaba, São Paulo State, Brazil; University of Perugia: Universita degli Studi di Perugia, ITALY

## Abstract

Currently available conventional breeding methods for broilers often result in impaired biomechanics and skeletal growth for the animals. The addition of environmental enrichment is an alternative which can help alleviate these effects. This study examines the effects of environmental enrichment on biomechanics, morphometry, and bone mass of broilers across various age groups. In total, 112 Cobb 500 chicks (50% male and 50% female) were used in a completely randomized design experiment, with 56 broilers per treatment (T1 and T2), carried out in subdivided plots. Each plot was subjected to a different treatment, as follows: all plots were subjected to the treatments (T1 = environmental enrichment and T2 = no environment enrichment) and the sub-plots held the broilers’ age groups (1, 7, 14, 21, 28, 35 and 42 days old). Eight broilers were euthanized on a weekly basis for two production cycles in order to perform morphometric (diameter and length) and biomechanical analysis of the response variables. These measurements were performed on the femur and tibia. Birds were subjected to classical linear fixed effects model and compared through Tukey’s mean test. Significant interactions between environmental enrichment and broiler age were noticed, particularly at 42 days, which displayed bone development for all variables under study. Except for the length of the femur of broiler chickens (p = 0.4638). Therefore, simple effects will not be evaluated. Environmental enrichment had a notable impact on tibia length (p = 0.0035), femur weight (p = 0.0014), and tibia weight (p<0.0001) at 42 days, indicating a favorable effect on skeletal growth in broilers. Enrichment resulted in a 1% increase in femur inertia, a 2% rise in tibia inertia, and a 1% enhancement in ultimate bending stress for both bones, displaying improved structural integrity and durability. Beneficial changes in bone morphology and biomechanics were observed at 42 days after enrichment.

## Introduction

Selection only focused on yield features in animal breeding can lead to animals’ health and well-being issues. Fast-growing broilers in poultry farming are susceptible to lameness, and it can be a significant source of death in comparison to rates recorded for slow-growing broilers. Genetic selection based on yield features has been successful in improving broilers’ yield; however, some support systems, such as the cardiovascular and skeletal ones, did not follow the achieved body mass increases, which made these birds more susceptible to suffer from skeletal system’s longer length or failure [1]. This outcome led to studies focused on finding strategies to strengthen broilers’ locomotor system in poultry farming [2].

The use of biomechanics in poultry farms is related to the effort types (compression, torsion, tension, shear and bending) bones are subjected [3]. Bone fracture cases happen when the load put over a certain region of the bone tissue exceeds its resistance [3, 4]. It is worth highlighting that sex, age, nutrition, the environment and hormonal balance are factors affecting bone features. Bone biomechanics studies applied to poultry farming mainly target the femur and the tibia, especially the last, given their relevance for poultry locomotion and support. It is so, because these bones are classified as too long for birds’ legs, since these bones are subjected to significant loads and stress during locomotion and weight support [3].

Environmental enrichment implementation in broiler chicken barns has been promising in improving bone quality, since several studies have shown that birds are encouraged to move around in enriched environments [5–8]. Broilers’ mobility leads to increased tibia mass, resistance to fracture, density and resistance [4, 9]. However, limited moves induce bone mass loss and reduce bone mechanical stability, besides impairing the locomotor bone’s biomechanical features in poultry [10, 11].

Many environmental types are applied in broilers’ production, such as hay bales [7, 12], perches [13, 14], ramps [12] and rice straw [8]. Specifically, the use of perches promotes natural behaviors and increases the well-being of animals in poultry farms, as well as having beneficial effects on reducing aggressive behaviors [13, 14]. It is also known that the use of the perch is used especially at night. The aim of the present research is to pick out an effective system that improve the bone-structure quality (femur and tibia) by encouraging broilers’ exercising by implementing environmental enrichment (perch). It must be done to reduce economic losses caused by bone fractures at birds’ capture, transport and slaughter, as well as to improve birds’ well-being. However, some questioning remains: are locomotor system bones of broilers in different age groups influenced by biomechanics and morphometry due to environmental enrichment? The herein advocated hypothesis states that birds subjected to environmental enrichment present bone strengthening in their first weeks of life.

The aim of the present study was to assess environmental enrichment adoption in poultry farming by using broilers in different age groups, as well as its influence on these birds’ locomotor system bones’ biomechanics, morphometry and weight.

## Materials and method

### Research regulation

The study was approved by the Ethics Committee on Animal Use (CEUA) of University of São Paulo–Luiz de Queiroz Agriculture School (USP/ESALQ), Piracicaba City, São Paulo State, Brazil, under protocol n. 2016/10. The study was carried out in this same higher education institution and was in compliance with the Animal Research: Reporting of *In Vivo* Experiments (ARRIVE) guidelines.

### Animals and management

A total of 112 Cobb 500 chicks (50% male and 50% female) were randomly allocated to two groups: T1 (environmental enrichment) and T2 (no environmental enrichment). The experiment took place in subdivided plots across age brackets (1, 7, 14, 21, 28, 35, and 42 days).

The birds living under a density of 15 cm^2^ per bird and 12 cm per perch (environmental enrichment). The birds were reared in a controlled environment and randomly distributed into 4 boxes (1.50m in length, 1.00m in width and 0.7m in height). The ground in each box was covered with rice straw and animals had access to water and feed, *ad libitum*. In total, 56 birds were subjected to each treatment (T1 = under environmental enrichment and T2 = lack of environmental enrichment, or conventional system)– 128 broilers were used for replacement purposes. Replacement birds were labeled with rings because they did not participate in the bone biomechanics analysis, and they were used as repetition over the whole experimental period, based on the methodology by Frutosa et al. [15] and Bang et al. [16]. Replacement birds were reared during two production cycles (42 days of life). They were subjected to the same rearing conditions during the experimental period (treatment, density, diet and weather). The experimental diets were formulated according to the strain manual recommendations. The bromatological composition of the ingredients of the feed formulations followed the recommendations of Rostagno et al. [17].

### Experimental design

Experimental design followed the completely randomized approach, with 8 repetitions (number of birds euthanized in different ages) and subdivided plots: plots held the treatments (T1 and T2) and subplots regarded broilers’ age groups (1, 7, 14, 21, 28, 35 and 42 days old). Data were evaluated using a classical linear fixed effects model (Eq 1), and Tukey’s test was utilized for mean comparisons.

$$Y_{ij}=\mu +\tau_{i}+e_{i}+\beta_{j}+{\left(\tau \beta \right)}_{ij}+\varepsilon_{ij}$$ (1)

Wherein, Y_ij_ = values observed for the i-th treatment and j-th subplot; μ = constant; τ_i_ = effect of the i-th factor A; e_i_ = plot residue; β_j_ = effect of the j-th factor B; (τβ)_ij_ = interaction between the i-th factor A and the j-th factor B; Ɛ_ij_ = subplot residue.

### Environmental enrichment

**Fig 1:**
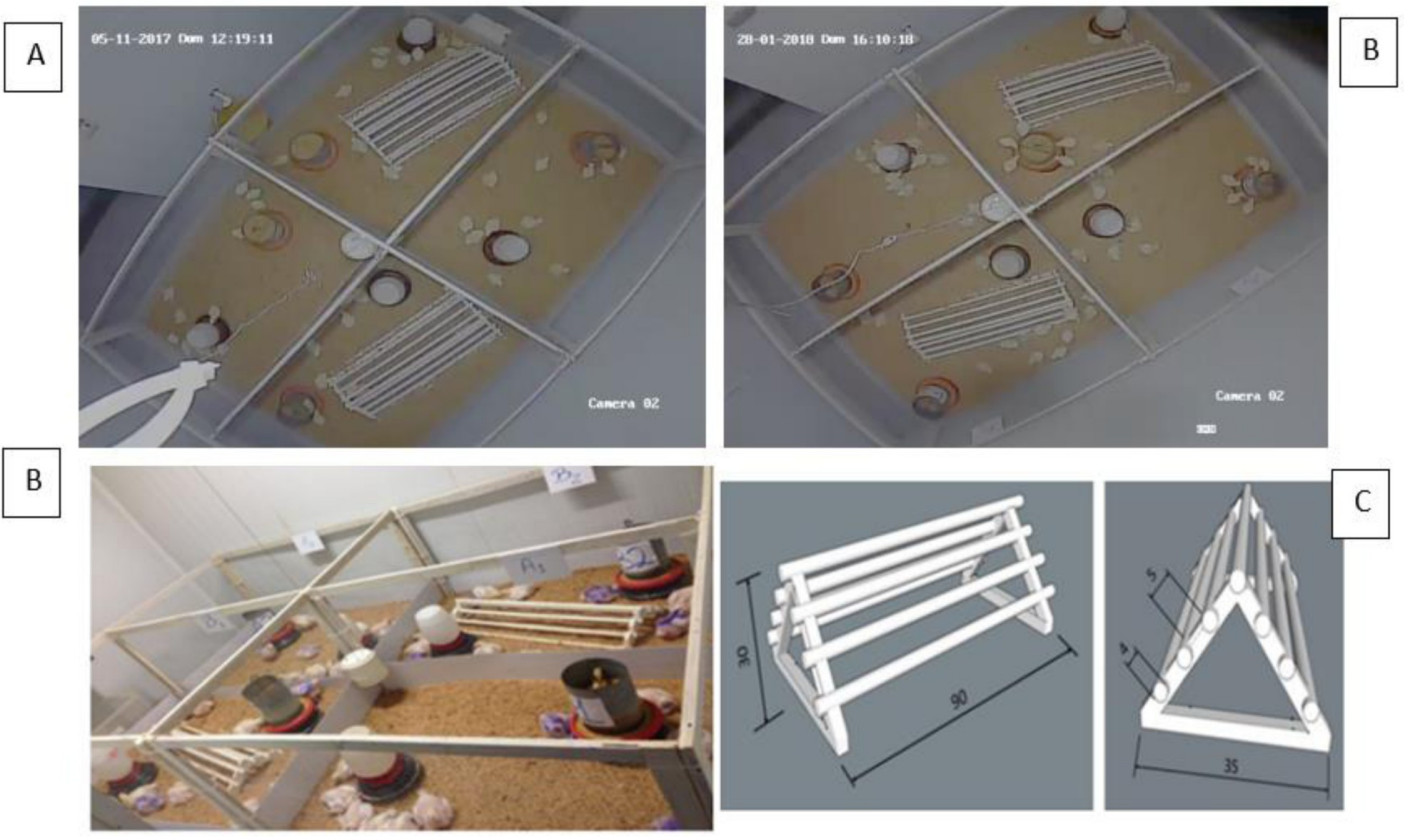
Aerial images (A) and boxes in the climatic chamber (B) and environmental enrichment model used in the research at the following dimensions: 90 cm in length, 35 cm in width, 30 cm in height, 4 cm in thickness and 5 cm spacing between stages (C) (Nazareno et al., 2022, 2024).

The herein suggested environmental enrichment model was based on a ramp with a perch on its top. This perch was an around-shaped stick made out of pine wood, which is a light material with low thermal conductibility ability—it favored thermal isolation. The model’s project was based on a perch placed 5cm from the ground, for a 1 to 21-day time-period and 10cm elevation from 22 to 42 days, based on Ohara et al. [12]. Occupation density of 15cm/bird and 4cm thickness were taken into account [18]. The following research dimensions were adopted to build the enriched environments: ramp with a perch on its top - 90cm in length, 35 cm in width and 10cm in height (Fig 1) [13, 14].

### Environment conditions

Microclimate variables (air temperature and relative humidity) were controlled with the aid of thermally isolated climatic chamber (controlled environment) with automatic temperature, relative air humidity, ventilation rate and light program control. According to Cobb [18], this equipment fulfills birds’ thermal-neutral needs. The light program set for the age group 0–35 days was 18 h light and 6 h dark, and that established for the group 35–42 days was16 h light and 8 h dark; ventilation rate was 0.04–0.21 m^3^s^-1^ per kg of bird. Yet, the controlled environment was added with two Hobo^®^ data loggers (model U12-012; Onset®, Piracicaba, Brazil) in order to ensure constant temperature and relative air humidity control (Table 1). Environmental conditions’ monitoring was periodically performed.

**Table 1:** Temperature and relative humidity of air ranges ideal for broilers in the current research based on Cobb (2012).

Age (days)	Air temperature (°C)	Relative humidity (%)
1	32–33	30–50
7	29–30	40–60
14	27–28	50–60
21	24–26	50–60
28	21–23	50–65
35	19–21	50–70
42	19–21	50–70

### Collected variables

**Fig 2:**
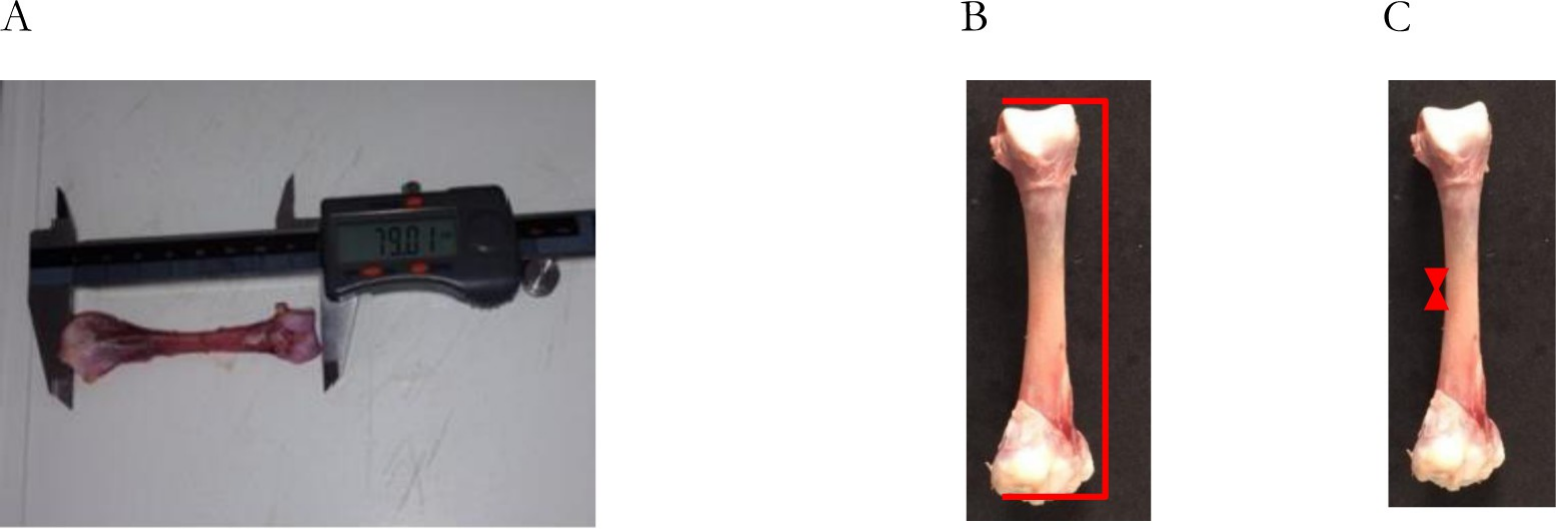
Images of length (A and B) and diameter (C) measurements taken of broilers’ tibia.

**Fig 3:**
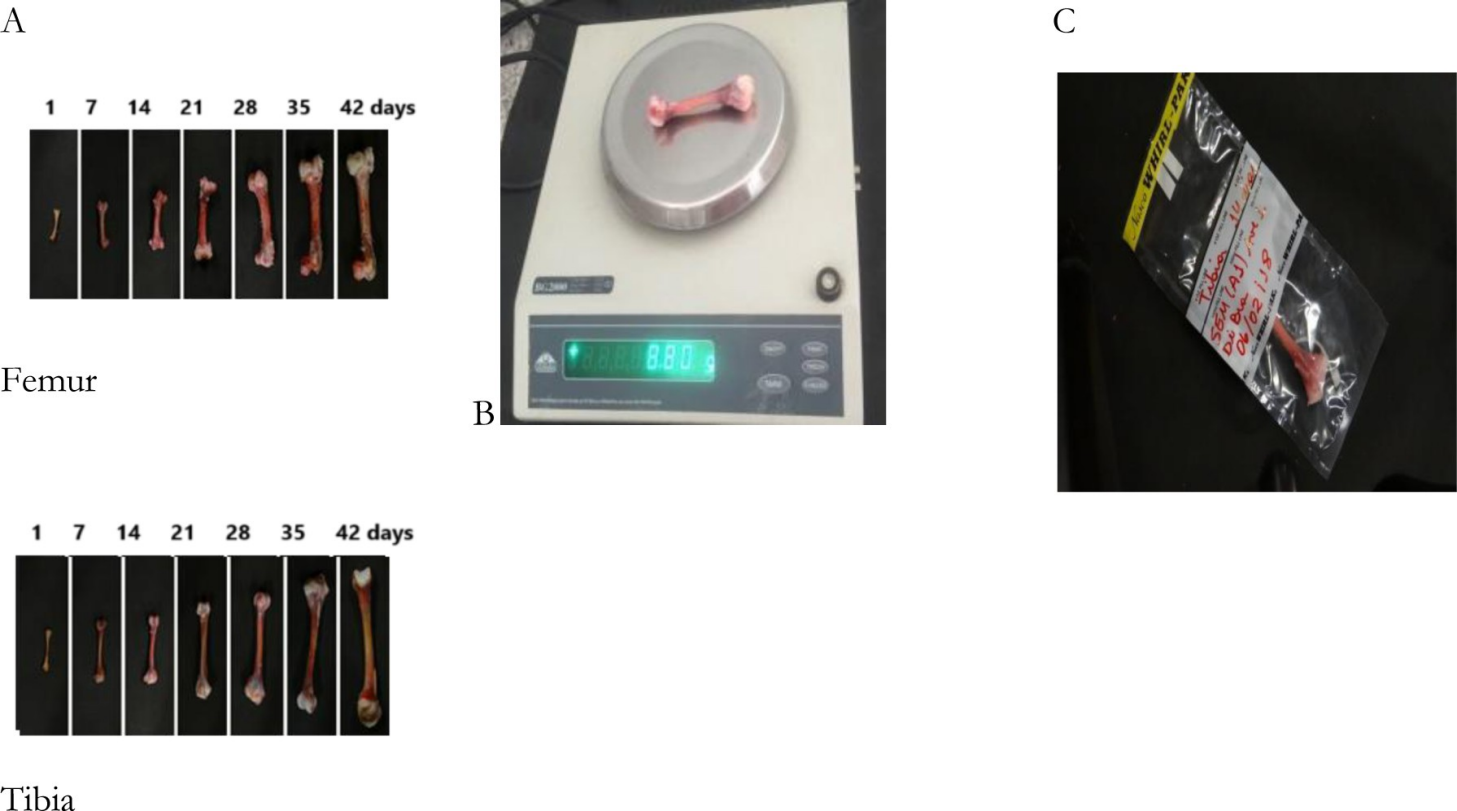
Images of different bone stages (A), tibia weight (B) and identification (C).

Eight (8) broilers were euthanized on a weekly basis for two production cycles in order to carry out the response variables, such as their bone morphometric and biomechanical analyses. Cervical dislocation was applied based on well-being standards. Subsequently, the right and left legs were removed through an incision performed with clinical scalpel. The right and left tibia and femur bones were removed after the aforementioned procedure and these bones were stripped and weighed. Subsequently, the morphometric properties of the tibias and femurs were measured and recorded: diameter (internal and external of the diaphysis of the ruptured section of each bone) and longitudinal length using a Digimess digital caliper with an accuracy of 0.02 mm (Fig 2). Bone weight measuring was carried out on semi-analytical scale BG2000, at 0.1g accuracy (Fig 3). All bones were identified separately, placed in plastic bags and stored at -20°C in the freezer (Consul) for the biomechanical analysis. Bones’ storage procedures and analyses were based on the methodology by Turner & Burr [19] and ASABE [20].

**Fig 4:**
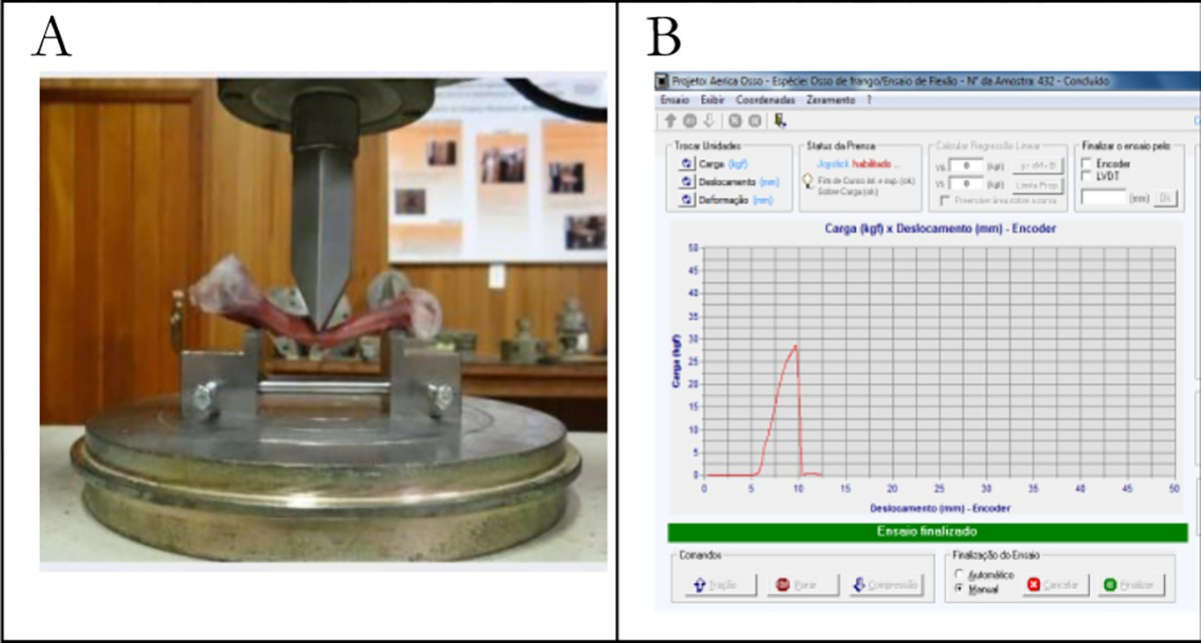
Images of broilers’ tibia biomechanical assessment with universal mechanical testing machine (A—moment of bone rupture in the ultimate bending strength) and force-deformation curve (B).

The biomechanical analyses of the right and left tibia and femur bones were carried out after the aforementioned procedures were done. The following biomechanical broiler bones’ features were assessed: applied force (N), initial cross-sectional area (cm^2^), inertia moment (10^−10^ m^4^) and ultimate bending stress (MPa). These features were measured through three-point flexion tests, with bones supported at their tips and mechanical load applied to the center of the diaphysis by using a universal mechanical testing machine, based on Turner & Burr [19] and ASABE [20]. The universal mechanical testing machine was coupled to a computer to continuously register data of both the applied mechanical load and the corresponding deflexing in a pair of points. These data generated the loading curving, which was used to set the mechanical parameters (Fig 4).

Load application rate set for the tibia and femur recorded constant speed of 10mm/min, based on ASABE [19] and Turner & Burr [20]. The distance between the two supports changed as broilers’ aged, and this outcome followed the tibia bones’ longitudinal length increase: 42 mm at 21 days, 48 mm at 28 days, 56 mm at 35 days and 66mm at 42 days [27]. Distance between the two supports in femur bones also changed as birds aged; it reached 30 mm at the age of 21 days and 40 mm at the ages of 28, 35 and 42 days–it followed bones’ longitudinal growth [26]. The literature did not provide the distance between the two tibia and femur supports at the ages of 1, 7 and 14 days; thus, it was necessary carrying out a previous correlation-based study to set these distances. Values recorded for the tibia were 17, 24 and 30 mm, whereas those recorded for the femur were 14, 17 and 24mm, at the age of 1, 7 and 14 days.

**Fig 5:**
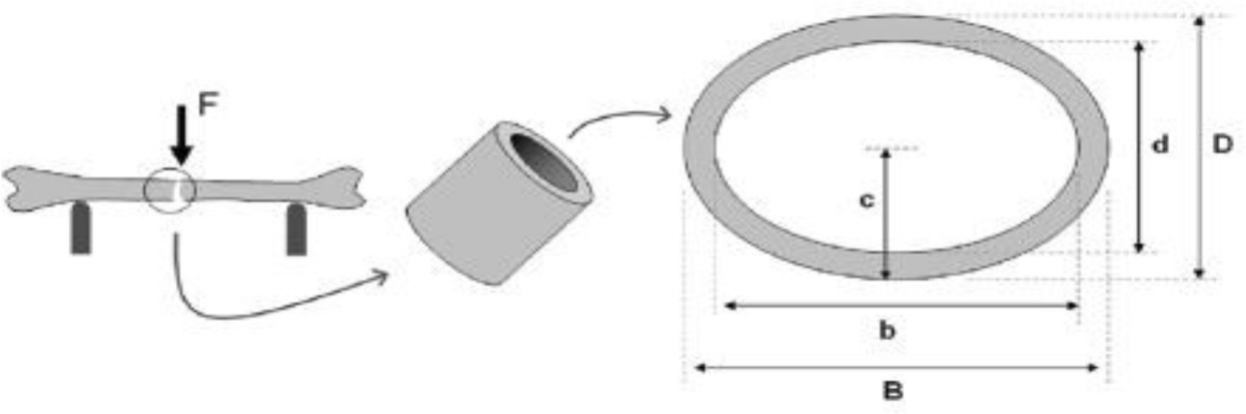
Representation of tibias’ cross-section as schematic hollow ellipse of the bones’ cross-section as ellipse quadrant (Reis et al., 2011).


Load assays and bone biomechanical parameters’ calculations were carried out based on inner and outer diameter measurements taken of the disrupted initial cross-sectional area of each bone, with the aid of digital caliper. Therefore, tibia and femur bones’ initial cross-sectional area was assumed with the aid of hollow ellipse [19, 20]. The initial cross-sectional area (A, cm^2^) was represented in Eq 2, inertia moment (I, m^4^) in Eq 3 and ultimate bending stress (σ, MPA) in Eq 4 [19, 20]. (Fig 5).

A=π[(B*D)−(b*d)] (2)

$$I=0.049[\left(B*D^{3})-(b*d^{3}\right)]$$ (3)

$$\sigma =\frac{\left(F*L*C\right)}{\left(4*I\right)}$$ (4)

Wherein,

B–outside major diameter (cm); b–inside major diameter (cm); D–outside minor diameter (cm); d–inside minor diameter (cm), corresponding to cross-section height based on its position in the flexing essay; F–applied force (N); L–distance between two points (cm); C–distance from neutral axis to outer fiber (cm)

### Statistical analysis

All response variables were investigated through ANOVA conducted based on the classic linear model of fixed effects. ANOVA assumptions were validated based on residue graphs, Shapiro-Wilk normality test and Hartley variance homogeneity. Treatments were compared to each other through Tukey’s mean test at 5% probability level. The data presented in the tables are the averages, coefficient of variation (%) and standard deviation. All analyses were carried out in the SAS statistical package [21].

## Results

Significant interactions between environmental enrichment and broiler age were noticed, particularly at 42 days, which displayed bone development (Tables 2–4) for all variables under study. Except for the length of the femur of broiler chickens (p = 0.4638; Data not presented). Therefore, simple effects will not be evaluated. Environmental enrichment had a notable impact on tibia length (p = 0.0035), femur weight (p = 0.0014), and tibia weight (p<0.0001) at 42 days, indicating a favorable effect on skeletal growth in broilers (Table 2). In addition, femur applied force presented a difference between T1 and T2 at the ages of 42 and 35 days, which reached the highest means: 331.5N and 329.5N, respectively. Nevertheless, broilers’ tibia applied force, the femur initial cross-sectional area and the tibia initial cross-sectional area only presented differences between T1 and T2 at the age of 42 days (Table 3). Finally, femur and tibia inertia were different between T1 and T2 at the age of 42 days: 4.97 10^−10^ m^4^ femur inertia and 3.35 10^-10^m^4^ tibia inertia. The femur initial cross-sectional areas of broilers’ and tibia were different between T1 and T2 at the ages of 42 and 35 days: 43.83 and 38.54MPa femur ultimate bending stress, and 71.78 and 64.79MPa tibia ultimate bending stress, respectively, this set of information reflects better structural integrity and durability (Table 4).

**Table 2:** Mean length and bone weight of tibia and femur of broilers subjected to T1 and T2 –with and without environmental enrichment, respectively.

• **Tibia length (mm)**							
Treatments	Birds’ age (days)						
	1	7	14	21	28	35	42
T1	32.04Ag	44.60Af	59.15Ae	75.12Ad	87.25Ac	100.02Ab	110.70Aa
T2	32.35Ag	44.71Af	62.50Ae	76.41Ad	87.88Ac	97.44Ab	108.37Ba
Treatment*Age (P-value)			0.0035				
Standard deviation			26.52				
Variation coefficient (%)			4.25				
• **Femur weight (g)**							
Treatments	Birds’ age (days)						
	1	7	14	21	28	35	42
T1	0.21Af	0.89Af	2.60Ae	5.33Ad	7.85Ac	11.07Ab	13.90Aa
T2	0.23Af	0.93Af	2.99Ae	5.47Ad	7.71Ac	10.56Ab	12.10Ba
Treatment*Age (P-value)			0.0014				
Standard deviation			4.50				
Variation coefficient (%)			18.03				
• **Tibia weight (g)**							
Treatments	Birds’ age (days)						
	1	7	14	21	28	35	42
T1	0.34Af	1.32Af	3.58Ae	7.55Ad	10.44Ac	15.00Ab	20.05Aa
T2	0.36Af	1.25Af	4.07Ae	7.71Ad	10.44Ac	13.72Ab	16.76Ba
Treatment*Age (P-value)			<0.0001				
Standard deviation			6.42				
Variation coefficient (%)			17.91				

^A-B^ Means with different uppercase letters in each column differ at 0.05 probability level in the Tukey ’s mean test (P<0.05)

^a–g^ Means with different lowercase letters in each line differ at 0.05 probability level in the Tukey ’s mean test (P<0.05)

**Table 3:** Means recorded for broilers’ locomotor system bones biomechanical features (applied force and initial cross-sectional area) subjected to T1 and T2 –with and without environmental enrichment, respectively.

• **Broilers’ femur applied force (N)**							
Treatment	Birds’ age (days)						
	1	7	14	21	28	35	42
T1	8.0Ae	42.4Ad	120.4Ac	215.8Ab	225.1Ab	329.5Aa	331.5Aa
T2	8.4Ae	35.8Ad	123.9Ac	194.6Ab	221.1Ab	261.8Ba	259.6Ba
Treatment*Age (P-value)			< 0.0001				
Standard deviation			32.15				
Variation coefficient (%)			17.60				
• **Broilers’ tibia applied force (N)**							
Treatment	Birds’ age (days)						
	1	7	14	21	28	35	42
T1	7.6Ad	27.2Ad	103.2Ac	211.1Ab	216.9Ab	231.8Ab	287.7Aa
T2	7.5Ac	25.0Ac	102.1Ab	172.8Ba	206.9Aa	208.1Aa	207.2Ba
Treatment*Age (P-value)			<0.0001				
Standard deviation			35.58				
Variation coefficient (%)			20.10				
• **Broilers’ femur initial cross-sectional area (cm**^**2**^**)**							
Treatment	Birds’ age (days)						
	1	7	14	21	28	35	42
T1	0.08Ae	0.24Ae	0.70Ad	1.12Ac	1.50Ab	1.54Ab	2.10Aa
T2	0.08Ad	0.25Ad	0.71Ac	0.10Ab	1.31Aa	1.60Aa	1.40Ba
Treatment*Age (P-value)			<0.0001				
Standard deviation			0.62				
Variation coefficient (%)			17.91				
• **Broilers’ tibia initial cross-sectional area (cm**^**2**^**)**							
Treatment	Birds’ age (days)						
	1	7	14	21	28	35	42
T1	0.08Ad	0.21Ad	0.56Ac	0.91Ab	1.16Ab	1.31Ab	1.62Aa
T2	0.08Ad	0.21Ad	0.60Ac	0,90Abc	1.13Aab	1.34Aa	1.28Ba
Treatment*Age (P-value)			0.0457				
Standard deviation			0.40				
Variation coefficient (%)			25.5				

^A-B^ Means with different uppercase letters in each column differ at 0.05 probability level in the Tukey ’s mean test (P<0.05)

^a–b^ Means with different lowercase letters in each line differ at 0.05 probability level in the Tukey ’s mean test (P<0.05)

**Table 4:** Means recorded for broilers’ locomotion system biomechanical features (inertia and ultimate bending stress) subjects to T1 and T2 –with and without environmental enrichment, respectively.

• **Broilers’ femur inertia (10**^**−10**^ **m**^**4**^**)**							
Treatment	Birds’ age (days)						
	1	7	14	21	28	35	42
T1	0.01Ad	0.05Ad	0.35Acd	0.94Ac	2.08Ab	2.32Ab	4.97Aa
T2	0.01Ac	0.05Ac	0.35Ac	0.79Ac	1.82Ab	2.85Aa	2.75Ba
Treatment*Age (P-value)			<0.0001*				
Standard deviation			0.10				
Variation coefficient (%)			29.51				
• **Broilers’ tibia inertia (10**^**−10**^ **m**^**4**^**)**							
Treatment	Birds’ age (days)						
	1	7	14	21	28	35	42
T1	0.01Ac	0.04Ac	0.34Ac	0.90Abc	1.34Ab	1.40Ab	3.35Aa
T2	0.01Ac	0.04Ac	0.24Ac	0.65Abc	1.35Aab	1.88Aa	2.05Ba
Treatment*Age (P-value)			0.0005				
Standard deviation			1.21				
Variation coefficient (%)			31.21				
• **Broilers’ femur ultimate bending stress (MPa)**							
Treatment	Birds age (days)						
	1	7	14	21	28	35	42
T1	27.38Ad	28.38Ad	29.31Ad	31.38Acd	34.32Ac	38.54Ab	43.83Aa
T2	27.89Ab	28.10Ab	28.89Ab	30.16Aa	32.53Aa	33.50Ba	33.60Ba
Treatment*Age (P-value)			<0.0001				
Standard deviation			25.49				
Variation coefficient (%)			27.34				
• **Broilers’ tibia ultimate bending stress (MPa)**							
Treatment	Birds’ age (days)						
	1	7	14	21	28	35	42
T1	34.64Ae	36.55Ae	38.68Ade	46.12Ac	52.10Ac	64.79Ab	71.78Aa
T2	30.01Ad	34.91Ad	37.79Ad	43.50Ac	52.76Abc	54.12Bb	62.52Ba
Treatment*Age (P-value)			0.0452				
Standard deviation			19.69				
Variation coefficient (%)			28.40				

^A-B^ Mean with different uppercase letters in each column differ at 0.05 probability level in the Tukey ’s mean test (P<0.05)

^a–e^ Mean with different lowercase letters in each line differ at 0.05 probability level in the Tukey’s mean test (P<0.05)

## Discussion

The present study is pioneer in assessing broilers’ locomotor system morphometric, biomechanical and bone weight features (femur and tibia) depending on birds’ age group. The main results in the current research are the first to disclose the beneficial effects of environmental enrichment as strategy to strengthen broilers’ bone structure at the age of 42 days, since it leads to birds’ well-being in industrial poultry farming and, consequently, reduces economic losses.

Environmental enrichment adoption has changed tibia length by 1%, femur weight by 1% and tibia weight by 1%, and such an increase in morphometry is associated with more exercising (motivation) practiced by birds subjected to stimulus in T1. According to Goff [22], more exercising leads to micro-fractures in the bones, and it increases the amount of minerals and the thickness of the bone matrix achieved during bone remodeling. It is important to point out that exercising increases the mechanical load on bone tissue due to muscle outer strengthening and contractions. This process helps remodeling the bones and increasing their weight [23].

Results in the current study meet those by Regmi et al. [3] and Rodriguez-Navarro et al. [4], who found that exercising stimulated bone and muscle formation in laying hens. Environment enrichment implementation boosts broilers’ will to move around; consequently, it improves the quality and the strength of their bones, besides increasing tibia weight and length [5, 6]. However, Karaarslan & Nazlıgül [24] did not find any influence of using perches in broiler barns on these birds’ tibia length.

Broilers’ locomotor system bones’ biomechanics was changed by environmental enrichment adoption; thus, it has also increased femur applied force by 1%, tibia applied force by 1%, femur initial cross-sectional area by 2% and tibia initial cross-sectional area by 1%, at the age of 42 days. The highest femur maximum strength and tibia recorded for the group subjected to environmental enrichment at the age of 42 days was closely related to these bones’ length and weight. The same was documented by Yalçin et al. [25], who found a positive correlation between bone weight and length. However, such an increase in the maximum strength does not imply improving the quality of the bone because this variable is closely related to bone geometry [26, 27]. According to Regmi et al. [3], increased exercising (motivation) by laying hens bred free in sheds has increased their bones’ maximum strength, cross-sectional area, weight and length (tibia and humerus). Birds’ low mobility tends to atrophy the bones and muscles due to their low use [4, 11, 28]. It is possible inferring that environmental enrichment is a sustainable option for bone strengthening [29, 30] by comparing results in the present research regarding fast-growth birds to those in the study by Damaziak et al., [1], who have shown that medium-growth hens could be bred up to 56 days without any risk of compromising their growth due to issues associated with bone-quality reduction in pelvic limbs.

Ohara et al. [12] assessed environmental enrichment based on using hay bales and perches in broiler barns and observed greater birds’ mobility (exercising). Karaarslan & Nazlıgül [27] found tibia weight increase and broilers’ exercising on perches. Yet, the adoption of this environmental enrichment also increased bone and muscle development in broilers [31].

The largest femur and tibia initial cross-sectional area were observed in the group of broilers subjected to enrichment, only at the age of 42 days, and it is related to bone weight. According to Leterrier et al. [32], there is close correlation between bone weight and the initial cross-sectional area. It is known that the larger the initial cross-sectional area the higher the amount of bone tissue. Therefore, these features make this structure more resistant to mechanical efforts [27, 33]. Thus, bone weight was influenced by exercising, and initial cross-sectional area enlarged due to this variable. Results recorded by Bizeray et al. [5] and Ventura et al. [6] corroborate the herein recorded ones.

Femur and tibia applied force, their initial cross-sectional area, inertia, ultimate bending stress and weight increased as broilers’ aged. Leterrier et al. [32] and Williams et al. [33] mentioned that birds’ age influenced bones’ biomechanical features, but they did not find mean values equivalent to the herein assessed age groups.

Bone inertia can get higher due to environmental enrichment (barriers and perches), since it can increase broilers’ mobility [6, 9]. However, low birds’ exercising throughout their development stage decreases their bone biomechanical features [4, 11]

Exercising leads to mechanical load increase, and it acts in bone tissues due to muscle contractions’ outer strength [3, 34]. Such an increase in mechanical load leads to tension strength, and it can increase bone resistance. Regmi et al. [3] observed weight gain, increased bone resistance and ultimate bending stress in the humerus and tibia of laying hens bred in barns. The aforementioned authors state that exercising was the factor improving these biomechanical features.

Using aerial perches increased tibia and humerus’ disruption strength in birds bred in cages with perches in comparison to those bred in conventional-system cages [35]. Therefore, more exercising, weight support and jumps on perches are factors capable of increasing bone mass [10], volume [36] and strength in birds. Foutz et al. [37, 38], in their turn, stated that low broilers’ exercising decreases tibia and humerus’ resistance to flexing, applied force, bone density and inertia.

Based on the results, environmental enrichment adoption increased broilers’ exercising. This process led to micro-fractures in locomotor system’s bones and, consequently, increased tibia and femur morphometric features and weight. This improvement in weight, length, applied force, initial cross-sectional area, inertia and ultimate bending stress was recorded at the age of 42 days.

Perches play a significant role in improving health conditions, behavior and welfare of birds, increasing the bone strength of the legs through continuous movements along the perch, as demonstrated in the results of our study. However, some studies report that their use can cause problems with traumatic injuries, producing fractures and an increase in the mortality rate [39]. However, it seems that these more moderate and severe injuries are more associated with metal perches compared to plastic perches [40] and softer materials [41]. In a complementary study using the same animals as in our study, but evaluating characteristics associated with physical integrity and keel and locomotor issues, we observed that the use of environmental enrichment favored the reduction of score 1 for plumage cleanliness and lameness in the animals. In addition, hock burn, foot dermatitis, and keel damage were not affected by the use of environmental enrichment [42]. Finally, we emphasize the importance of carrying out an economic analysis study represented by the costs required for the application of such enrichment system, which is difficult to apply in all types of systems and, therefore, its use requires a careful cost-benefit analysis.

## Limitation

Our results demonstrated that the use of perches as environmental enrichment improved the parameters of biomechanics and bone morphology, mainly attributed to the increase in physical activity of animals that underwent environmental enrichment. However, a limitation of this study is that we did not measure the activity of birds using the perches. What we know is that birds subjected to environmental enrichment showed greater physical activity [14]. Another limitation is that we did not evaluate the identification of bone tissue microfractures in conjunction with the bone biomechanics data. We encourage further studies on environmental enrichment’s effects on bone biomechanics, morphological parameters, behavioral responses, and the identification of bone tissue microfeatures, analyzed simultaneously to verify the potential interrelationships among these parameters.

## Conclusion

Beneficial changes in broilers resulting from environmental enrichment adoption were recorded at the age of 42 days. These results indicate that integrating environmental enrichment in broiler production could improve skeletal health and overall welfare, potentially resulting in stronger and healthier birds.

## Supporting information

S1 File (XLSX)
